# Specificity of weightlifting bench exercises in kayaking sprint performance: A perspective for neuromuscular training

**DOI:** 10.3389/fphys.2022.898468

**Published:** 2022-07-22

**Authors:** Cristian Romagnoli, Giorgio Gatta, Niloofar Lamouchideli, Antonino Bianco, Stefano Loddo, Anas R. Alashram, Vincenzo Bonaiuto, Giuseppe Annino, Elvira Padua

**Affiliations:** ^1^ Department for Life Quality Studies University of Bologna, Bologna, Italy; ^2^ Sport Engineering Lab, Department Industrial Engineering, University of Rome “Tor Vergata”, Rome, Italy; ^3^ Department of Human Neuroscience, Faculty of Medicine and Dentistry, Sapienza University of Rome, Rome, Italy; ^4^ Sport and Exercise Sciences Research Unit, Department of Psychology, Educational Science and Human Movement, University of Palermo, Palermo, Italy; ^5^ Italian Canoe/Kayak Federation (FICK), Rome, Italy; ^6^ Department of Physiotherapy, Isra University, Amman, Jordan; ^7^ Department of Medicine Systems, University of Rome “Tor Vergata”, Rome, Italy; ^8^ Centre of Space Bio-Medicine, “Tor Vergata” University of Rome, Rome, Italy; ^9^ Department of Human Science and Promotion of Quality of Life, San Raffaele Open University of Rome, Rome, Italy

**Keywords:** optimal load, power-based training, sprint performance, propulsive force, resistance training

## Abstract

Several studies showed significant differences between bench lift exercises without investigating which is more related, in biomechanical and neuromuscular terms, to improve the sprint flatwater kayak performance. This study aims to compare the power-load and velocity-load neuromuscular parameters performed in prone bench pull (PBP), and bench press (BP) exercises to identify which of them meet the gesture specificity in sprint flatwater kayak performance. Ten elite kayakers participated in this study. Power-load, velocity-load relationships, the maximum dynamic strength, and the kayak sprint performance test were assessed. The power-load and velocity-load relationships showed significant differences between the PBP and BP for each considered load. The kayakers showed a significant correlation between maximum power performed on the PBP and the maximum velocity reached in the kayak sprint (r = 0.80, *p* < 0.01) and the stroke frequency (r = 0.61, *p* < 0.05). Conversely, the maximum power performed on the BP did not correlate with the kinematic parameters analyzed. In addition, the maximum dynamic strength in the PBP and BP did not correlate with the maximum velocity and stroke frequency. Furthermore, no significant difference was observed in both the bench exercises for the maximum dynamic strength (*p* > 0.05). The results of this study suggest that the maximal muscular power expressed in PBP exercise only seems to be more specific in kayak velocity performance compared with maximal dynamic strength and with all dynamic parameters recorded in the BP. This will allow coaches and trainers to use specific bench exercises for specific neuromuscular kayakers’ adaptations during the whole competitive season.

## Introduction

Canoeing/kayaking is a sport where upper limbs are predominant in the propulsion phase of the boat while the action of lower limbs counteracts only the consequent kayak rotations (Mann and Kearney, 1980; Begon et al., 2010; Baker, 2012) even though in recent studies were observed a connection between improvements in lower limbs force and kayak sprint velocity (Nilsson and Rosdahl, 2016; Lum and Aziz, 2020). To maximize kayak velocity, the paddler generates high propulsive power by applying forces on the paddle blade during each stroke (Aitken and Neal, 1992; Michael et al., 2008). During the race, the kayak shows a changeable velocity (ranging from 4.63 to 5.38 m/s) generated by the paddler’s actions against the drag forces (Kendal and Sanders, 1992; Zumerchik, 1997; Gomes et al., 2015b). Therefore, to increase the kayak velocity, the paddler, dipping and pulling backward the blade (pull phase), has to produce a propulsive force more significant than the drag force (Millward, 1987; Jackson, 1995). Differently, during the aerial phase, only the drag forces (friction, form, wave) act on the kayak, decelerating it (Bonaiuto et al., 2020). Therefore, it is possible to deduce that the kayak means velocity is the consequence of the combined effects of the propulsion and the drag forces (Pendergast et al., 2005; Michael et al., 2009). In order to improve the propulsion phase useful to reduce the race time performance, the kayaker usually conditions the strength and power of upper limbs muscles through the prone bench pull (PBP) and bench press (BP) exercises (Akca and Muniroglu, 2008; García-Pallarés et al., 2009; Pearson et al., 2009; McKean and Burkett, 2010, 2014; Ualí et al., 2012; Hamano et al., 2015; Bielik et al., 2017; Bjerkefors et al., 2018; Winchcombe et al., 2019). Uali et al. (2012) reported that heavy resistance training performed in bilateral bent pull and one-arm cable row significantly correlated with the start phase of kayak sprint performances. In addition, Liow and Hopkins (2003), using both bench press and bilateral dumbbell prone lifts exercises, have shown that heavy resistance training seems to be more effective in conditioning the start phase (0–15 m) of kayak sprint performance while an explosive power training (low loads performed at high contraction velocity) could be more effective to maintain kayak velocity. However, it is necessary to consider that BP and PBP exercises present some distinctive biomechanical and neuromuscular features that make them antagonistic exercises to each other (Pearson et al., 2009; Sánchez-Medina et al., 2014). In this context, it should be more appropriate to consider these differences in specific strength and power conditioning and assessments in those sports disciplines that use upper limbs differently in pushing or pulling actions (Sánchez-Medina et al., 2014). According to these considerations, to increase the propulsive power produced by the paddler, it is necessary to condition in a dry-land environment, specific kinetic muscle chains and neuromuscular patterns using the power based-training method (Cronin et al., 2001). For that, it is essential to determine the power-load and velocity-load relationships, analyzed on bench exercises, monitoring the kinetic parameters with a dynamometer during an increasing loads test performance (Pearson et al., 2009; Sánchez-Medina et al., 2014; Sreckovic et al., 2015). Thus, this study aims to compare the power-load (p-l) and velocity-load (v-l) relationships expressed in BP and PBP exercises, verifying which of their dynamic parameters, 1RM and maximum power (P_max_), is more correlated with the maximum velocity reached during flatwater kayak performance.

## Materials and methods

### Participants

Ten elite male kayak athletes [age: 28.88 ± 2.26 (yrs), height: 1.85 ± 0.04 (m), weight: 84.93 ± 5.96 (kg), Body Mass Index: 24.60 ± 1.46 (kg/m^2^)] were involved in the study. They are members of the Italian Federation Canoe-Kayak (FICK) team with wide experience in international competitions. The subjects trained ten times a week during the study period (May), including four dry-land training and seven water sessions. The study was reviewed and approved by the Internal Research Board of “Tor Vergata” University of Rome. The subjects provided their written informed consent to participate in this study. All procedures were carried out in accordance with the Declaration of Helsinki.

### Testing procedure

In order to determine 1RM, power-load, and velocity-load relationships, a linear encoder (Bosco et al., 1995) was used during the increasing load test performed on the PBP and BP exercises. Regarding the water tests, three trials of the 50 m all-out kayak sprint test (KST) were assessed to measure maximum velocity and stroke frequency. Each athlete was evaluated in five sessions (1RM_PBP_, 1RM_BP_, PBP_p-l & v-l_, BP_p-l & v-l,_ and KST assessment) separated by 24 h of rest for each load test while 48 h of rest between the last load session and KST.

### Power-load and velocity-load relationships

Before the strength and power tests, the athletes performed a warm-up for the upper limbs completed in 20 min (5–8 min for static/dynamic stretching and joint mobilization) and 5–12 min for shoulder circumduction and shoulder horizontal abduction and adduction).

The standard procedure to assess power-load, velocity-load relationships, and maximum strength through the 1RM were determined in the PBP and BP exercises as suggested by Sreckovic et al. (2015).

In the PBP exercise, a modified Smith machine was used (Pearson et al., 2009), where the subject was in the prone position on the bench, grabbing the barbell positioned on two fixed lateral supports. The pull phase started with both elbows in full extension. After that, it was requested to the athlete the maximum effort to reach with the barbell the lower part of the bench (the thickness of the bench from the top of the padding to the bottom of the bench was 8 cm) without lifting the chest from the horizontal plane. Furthermore, during the pulling action, the athletes were not allowed to use their lower limbs to hold on to the bench. Conversely, during the BP, the subject was supine on the bench with their head supported. The barbell initially positioned at the same chest level (resting on fixed supports) was pushed upward as fast as possible up to the maximum extension. The subjects were not allowed to bounce the barbell off the chest or lift the shoulders or trunk off the bench. Only the concentric actions (pushing for BP and pulling for PBP) were assessed in the present study with a linear encoder. During each test, the athletes observed 4 minutes of passive recovery time for each lifted load. The increasing loads’ lift was selected in 20–40–60–80–100% of 1RM for each athlete. The test of 1RM was carried out with an accuracy of 5 kg.

### Kayak sprint test

Each KST trial, performed in a single session, in calm water with no influence of currents and with absent or negligible wind velocity conditions, was preceded by a standard warm-up phase where the kayaker performed 10 min of continuous paddling at moderate pace velocity followed by five trials of 50 m at increasing velocity (near to maximum velocity), observing 3 min of rest between each sprint trial.

The KST consists of three trials of 100 m each where the first 50 m were covered increasing velocity gradually up to maximum and performing the last 50 m at all-out pace velocity. Between the trials, the athletes observed 5 min of rest. The velocity was measured by the E-Kayak system (Bonaiuto et al., 2020), which was placed behind the paddler’s seat with the GPS antenna positioned over the boat to obtain the best signal strength. The best sprint performance was selected for statistical analysis.

## Statistical analysis

The statistical analysis was performed using SPSS software version 20.0 (SPSS, Chicago, I). The normality of each variable was initially tested with the Shapiro Wilk test, and all the variables presented a normal distribution. Standard statistical methods were used to calculate the mean values, the standard deviations (SD), and the 95% confidence intervals for the mean (95% CI). In order to verify the correlation between maximum power, one RM, and kinematic parameters of sprint kayak (maximum velocity, stroke frequency), the Pearson product-moment correlation coefficient (r) was used. The repeated measure ANOVA (between-subjects factor) was used to evaluate the differences between BP and PBP exercises. Bonferroni corrected post-hoc analysis with paired measure was used. Statistical significance was accepted at *p* < 0.05.

## Results

The repeated measure (between-subjects factor) showed a significant difference between the values of power and velocity in PBP and BP for loads ranging from 20 to 100% of one RM, as reported in Table 1.

**TABLE 1:** Comparison between values of Power (W) and Velocity (m/s) expressed during BP and PBP. All values are mean ± SD (95% Confidence Interval). Significant difference (between subject) as reported for *p* < 0.05 (*), for *p* < 0.01 (**) and for *p* < 0.001 (***).

Parameters	Bench press	95% CI	Prone bench pull	95% CI	*p*
Power _20%_	319.13 ± 129.40	226.56–411.71	484.44 ± 66.56	436.82–532.06	**
Power _40%_	497.09 ± 202.62	352.15–642.04	720.99 ± 62.18	676.51–765.47	**
Power _60%_	548.00 ± 218.65	391.58–704.42	805.16 ± 75.52	751.13–859.19	**
Power _80%_	471.84 ± 177.83	344.63–599.06	736.95 ± 82.67	677.81–796.09	***
Power _100%_	268.63 ± 93.93	201.43–335.82	516.36 ± 91.05	451.22–581.49	***
Velocity _20%_	1.16 ± 0.35	0.91–1.42	1.80 ± 0.24	1.62–1.98	**
Velocity _40%_	0.93 ± 0.27	0.73–1.12	1.45 ± 0.18	1.32–1.58	**
Velocity _60%_	0.69 ± 0.19	0.55–0.83	1.09 ± 0.12	1.01–1.18	**
Velocity _80%_	0.45 ± 0.12	0.36–0.53	0.75 ± 0.06	0.70–0.79	***
Velocity _100%_	0.21 ± 0.06	0.17–0.25	0.39 ± 0.04	0.36–0.43	***

**FIGURE 1:**
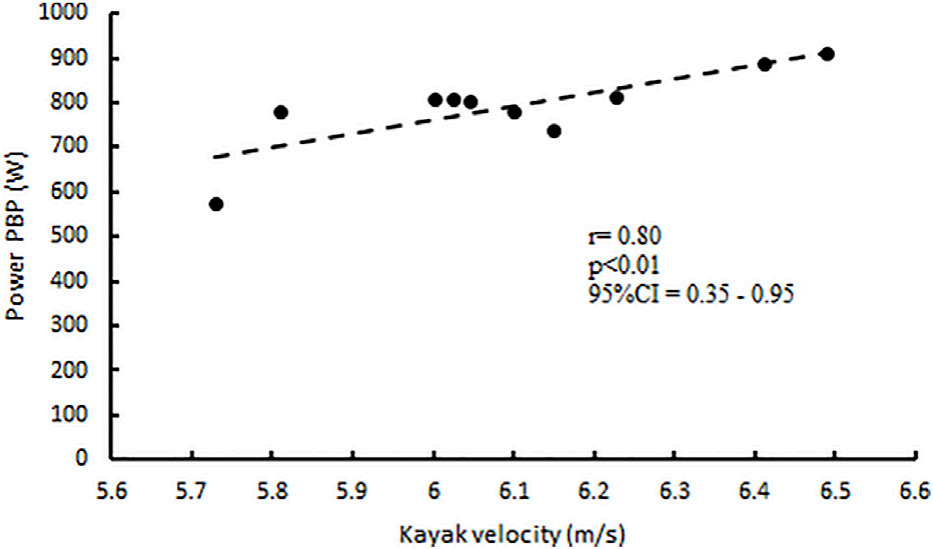
Correlation between P_max_ (W) expressed at PBP, and kayak velocity (m/s) reached on KTS.

The linear regression between the muscle P_max_ expressed in the PBP and the average velocity during the all-out pace 50 m KST has shown a close relationship with r = 0.80 and *p* < 0.01 (Figure 1; Table 2), while the correlation with stroke frequency shows an r = 0.61 and *p* < 0.05 (Table 2).

**TABLE 2:** Dynamic parameters (1 RM and P_max_ obtained for each athlete) expressed at PBP and BP in correlation with kinematic parameters observed on KTS. All values are mean ± SD. Correlation (95% Confidence Interval) is reported *p* < 0.05 (*) and *p* < 0.01 (**).

Variables	Mean ± SD	Correlation with KST_(50m)_ velocity (m/s)	95% CI	Correlation with stroke frequency (stroke/min)	95% CI
BP					
1RM (kg)	135.50 ± 13.86	0.46	−0.23–0.85	0.12	−0.51–0.73
P_max_ (W)	549.50 ± 219.32	0.12	−0.55–0.69	0.02	−0.23–0.85
PBP					
1RM (kg)	137.50 ± 12.52	0.33	−0.38–0.79	0.08	−0.52–0.71
P_max_ (W)	811.07 ± 70.89	0.80**	0.35–0.95	0.61*	−0.02–0.89

**FIGURE 2:**
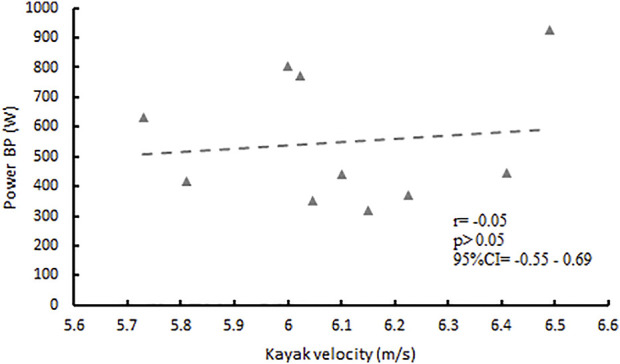
Correlation between P_max_ (W) expressed at BP, and kayak velocity (m/s) reached on KTS sprint.

Conversely, a poor correlation has been found between the P_max_ in BP exercises and the velocity measured during KST with r = 0.12 and *p* = 0.74 (Figure 2; Table 2).

In contrast to the Pmax, the 1RM showed a poor or no significant correlation with the average velocity and paddle stroke frequency during the KST (Table 2).

## Discussion

Both the power-load and velocity-load relationships, carried out by this study, respectively maintain the same quadratic and linear trend (Jaric, 2015; Sreckovic et al., 2015), such as those observed in the leg or arm extensors muscles during the use of isotonic (Bosco et al., 1995) or isokinetic devices (Perrine and Edgerton, 1978) or ballistic movements (Bosco and Komi, 1979; Pearson et al., 2009; Sánchez-Medina et al., 2014). In accordance with previous studies, in BP exercise the load to perform maximum power was 60% of 1RM (Izquierdo et al., 1999, 2002; Cronin et al., 2001). Conversely, in the PBP, our findings (60% of one RM) show values less than those reported in the literature (70–80% of 1RM) (Pearson et al., 2009; Sánchez-Medina et al., 2014).

The resultant curves for PBP and BP exercises show a significant difference between the kinematic and dynamic parameters for each lifted-up load considered in this study (Table 1). Probably, these differences in results could be associated with the different muscular kinetic chains involved in both bench lift test performances (Costill et al., 1976). In fact, from an anatomical point of view, the muscles involved as prime movers in the PBP exercises (i.e., latissimus dorsi, biceps brachialis, and brachialis) are composed of longer muscle fibers with a reduced angle relative to the force-generating axis (pennation angle) with a consequent faster muscle contraction than those involved as prime movers in the BP exercises (i.e., pectoralis major and triceps brachialis) which are characterized by shorter fibers and a greater pennation angles (Lieber and Fridén, 2000; Pearson et al., 2009). This allows generating more force as a consequence of slower muscle contraction velocity. Considering the muscle’s involvement during water paddle performance, Logan and Holt (1985) analyzed, in addition to the upper, lower limbs, and pelvis musculature, the detailed activation of back muscles during each subphase of the paddle stroke cycle. Subsequently, Trevithick et al. (2007) showed that the supraspinatus, the upper trapezius, the latissimus dorsi, serratus anterior, and the rhomboid major show a consistent activity pattern during kayak stroke. Moreover, it has been observed that the activity of the latissimus dorsi increases during the pull phase in water and reaches its peak during the following intermediate phase, confirming its role as a prime mover muscle during the in-water phase of the paddle stroke (Yoshio et al., 1974; Trevithick et al., 2007; Fleming et al., 2012). Based on these considerations, the PBP exercise seems more specific than BP ones relative to the technical paddle gesture (Sánchez-Medina et al., 2014).

Several studies have verified a positive correlation between strength profile and kayak performance, using isometric strength tests performed in BP and PBP and kayak ergometer performance (Lum and Aziz, 2020; Petrović et al., 2021). Nevertheless, these studies have not considered other relevant dynamic and kinematic parameters involved in both bench lift exercises and flatwater kayak performances; in contrast to the studies mentioned above, our findings have shown a poor correlation, for each bench lift exercise, between the maximum dynamic strength (1RM) and the maximum kayak velocity (Table 2). Conversely, only the PBP maximum power is significantly correlated with the maximum kayak velocity performance (r = 0.80; *p* < 0.01) (Figure 1) and the related stroke frequency (r = 0.61; *p* < 0.05) (Table 2). Probably, the differences found between this study and the previous ones (Lum and Aziz, 2020; Petrović et al., 2021) could be due to the different dry-land test protocols performed (dynamic Vs. isometric) and kayak test performance (50 m sprint flatwater kayak Vs. kayak ergometer). On the contrary, the data obtained from the BP exercise show a poor correlation (Figure 2; Table 2) not only between 1RM but also with the P_max_ and the kinematic parameters of the KST analyzed, showing the scant specificity with the biomechanical parameters and the muscular kinetic chains involved during the paddle stroke.

Differently, in agreement with the principle of specificity and training monitoring (Sale and MacDougall, 1981), only the P_max_ developed in the PBP exercise is coherent with the technical paddle gesture (Jackson et al., 1992; Tzabiras et al., 2010; Gomes et al., 2015a). Thus, in accordance with other factors such as paddling techniques, athlete-canoe interactions, and environmental conditions, the maximal muscle power output of the upper limbs seems to play an essential role as a limiting factor of the flatwater kayak performance.

### Limitations

This study has some limitations related to the kayak sprint distance considered and, even though negligible, the exact knowledge of environmental parameters (wind velocity, water temperature, and current velocity). Furthermore, the lack of reliability data due to the trial period being too close to the start of international competitions which, given the observational nature of this study and the high reliability observed for this test in other studies, should not significantly affect the observed differences in both bench tests. Finally, only two upper limb exercises and no lower limb exercises were considered.

## Conclusion

In conclusion, the PBP exercise seems to show a superior biomechanical and neuromuscular coherence with technical paddle gestures than BP.

For opposite reasons, the latter exercise is advisable as a complement to the conditioning of the antagonist’s muscles due to their synergistic role played during the aerial phase of the counter-lateral arm during the propulsive water phase of the paddling.

Future studies must investigate the effect of PBP and BP on 200–500 and 1000 m. Moreover, to understand if a power-based training rather than maximal force training before the competitions could be helpful in the kayak performance improvement.

## Data Availability

The raw data supporting the conclusion of this article will be made available by the authors, without undue reservation.
